# Younger Age Is an Independent Predictor for Poor Survival in Patients with Signet Ring Prostate Carcinoma

**DOI:** 10.1155/2011/216169

**Published:** 2010-07-20

**Authors:** Jue Wang, Fen Wei Wang, George P. Hemstreet

**Affiliations:** ^1^Department of Internal Medicine, Section of Oncology-Hematology, University of Nebraska Medical Center, Omaha, NE 68198-7680, USA; ^2^Department of Internal Medicine, Creighton University School of Medicine, Omaha, NE 68131, USA; ^3^Urologic Surgery Section, Department of Surgery, University of Nebraska Medical Center, Omaha, NE 68198-2360, USA

## Abstract

*Objective.* The aim of this study was to examine the epidemiology, natural history, treatment pattern, and predictors of long-term survival of signet ring prostate carcinoma (SRPC) patients based on the analysis of the national Surveillance, Epidemiology, and End Results (SEER) database. *Methods & Results*. Between 1980 and 2004, a total of 93 patients with pathologically confirmed SRPC were identified. The mean age was 70 ± 11 years old. 82.8% of the patients had poorly or undifferentiated histology grade. 13.9% patients presented with metastatic disease. The 1-, 3-, and 5-year cancer-specific survival rates were 94.6%, 89.6%, and 83.8%, respectively. Using multivariate Cox proportional hazard model, younger age (40–50 versus age >70 yrs, *P* = .01), advanced tumor stage (distant versus local/regional, *P* = .02), and earlier diagnosis year (before 1995 versus after 1995, *P* = .01) were predictors of worse cancer specific survival. *Conclusions.* Despite more aggressive cancer therapy, younger SRPC patients had a worse cancer specific survival. This information could be useful when counseling these patients and emphasizes the need for new strategies and molecular-based therapeutic approaches for younger patients with SRPC.

## 1. Introduction

Since the first description by Giltman in [1], approximately 100 cases of prostate signet ring cell carcinoma (SRCC) have been reported. One previous report reviewed 200 cases of prostatic adenocarcinoma; the authors identified five cases (2.5%) with signet ring cell differentiation [2]. Signet ring cells are frequently seen as a minor population in high grade adenocarcinomas, while the pure form of this entity is extremely rare [3]. Microscopically, signet ring cells are characterized by a clear cytoplasmic vacuole eccentric nucleus. These cells may be arranged in sheets, small clusters, or as dispersed single cells. The tumor cells are positive for both prostate-specific antigen (PSA) and prostate-specific acid phosphatase (PAP). A minority of cells may stain weakly positive for mucin. These characteristics can be used to differentiate them from intestinal tumor [4]. Saito and Iwaki [5] reviewed all the mucin-producing adenocarcinomas of the prostate reported in the English literature and described three subtypes: (1) mucinous carcinoma; (2) signet ring cell carcinoma, defined as having signet ring morphologic structure in at least 25% of the tumor volume; (3) mucinous carcinoma with signet ring cells, defined as having extracellular mucin in at least 25% of the tumor volume or having signet ring morphologic structure in less than 25% of the tumor volume. 

Thus, signet ring prostatic carcinoma (SRPC) is a rare form of adenocarcinoma. Although several small case series have been reported, most of these reports were retrospective reviews from single institutional experiences and focused on the histopathology [2, 6–10], and there are even fewer reports concerning treatment outcomes and followup. The demographic characteristics and clinical sequella of SRPC remains ill-defined, and patients' outcomes are inconsistent among studies [1, 5, 11]. The clinical significance and biologic behavior of this subgroup of primary prostate carcinomas need to be further characterized by performing more extensive studies with larger sample size and long-term followup. The aim of this study is to examine the demographic and clinical characteristics of patients with SRPC identified through the Surveillance, Epidemiology, and End Results (SEER) Program database and to determine the prognostic factors impacting cancer-specific survival.

## 2. Methods

### 2.1. Data Source

SEER retrieves patient records from multiple locations across the United States and is regarded as a model population-based tumor registry. SEER 9, 13, and 17 registries cover approximately 9.5%, 13.8%, and 26.2% of the total US population, respectively. In this study, we used the SEER data based on the November 2006 submission. Data for this study were obtained from SEER* Stat public-use data files, available on internet or CD-ROM from the National Cancer Institute [12].

### 2.2. Study Population

The cases of SRPC were extracted from the SEER on the basis of anatomic site and histology type. All patients over the age of 18 years diagnosed with SRPC in the SEER national cancer registry between January 1980 and December 2004 were evaluated. Cases identified at the time of autopsy or by death certificate only and patients with more than one primary tumor were excluded from the survival analyses.

### 2.3. Variables

Patients' social demographic characteristics (i.e., age, race/ethnicity, and marital status) and tumor grade and stage at the time of diagnosis were determined from the SEER database. SEER general summary stage [12] classifies patients as having local, regional (extension into adjacent tissues or nodal involvement), or distant disease. The World Health Organization's standard grading system was used with four separate categories (well, moderately well, poorly differentiated, and undifferentiated). For prostate cancer cases, SEER database recorded the highest value of PSA tests at the time of diagnosis under the variable named “Tumor Marker 2”. In the database, PSA was categorized as (a) none, (b) positive, (c) Negative, (d) borderline, undetermined whether positive or negative, (e) ordered, but results not in chart, and (f) unknown or no information [12]. According to the available information, we grouped PSA levels into three groups in this study: above normal (positive), normal (negative), or unknown.

### 2.4. Statistical Analysis

Age-adjusted incidence rates and their 95% CIs were calculated for SRPC in all patients, in men and women separately and in each of the 3 broad race categories (whites, blacks, and others). For calculation of the age-adjusted incidence rates, the US general population for the year 2000 was used as a standard population. 

Discrete data are reported as frequencies and compared by chi-square and Fisher's exact tests as appropriate. Continuous data are reported as mean ± SD and compared by student's *t*-test. Multivariate logistic regression analyses were used to determine the factors associated with receipt of radical prostatectomy and radiation therapy. Survival duration was measured by the Kaplan-Meier method and compared by the log rank test. Multivariable Cox proportional hazards model was used to identify independent predictors of long-term cancer-specific death. 

SEER*Stat 6.2.4 (Surveillance Research Program, National Cancer Institute) was used for incidence and limited-duration prevalence analyses. All other statistical calculations were performed by SPSS 12.0 (Apache Software Foundation 2000). Comparative differences were considered statistically significant when the *P*-value was <.05.

## 3. Results

### 3.1. Frequency and Incidence

A total of 588,101 patients with prostate cancer were identified in the SEER 17 registries between January 1980 and December 2004. When we restricted the search to signet ring cell carcinoma histology, a total of 93 patients were identified, representing 0.02% of all patients with prostate cancers. 

Using linked population files, the incidence of SRPC as a rate per 100,000 per year, age-adjusted to year 2000 US standard population was calculated. An age-adjusted incidence of 0.0088 per 100,000 was observed in the study periods. Detailed incidence data by time period, gender, and race are included in Table 1.

### 3.2. Patient and Tumor Characteristics

Table 2 provides a detailed demographic, tumor characteristics, and treatment information on 93 patients with SRPC. Of the 93 patients with SRPC identified in the SEER database during the study period, the mean age of the cohort was 70 ± 11 years old (median age, 69 years, with a range of 40 to 92 years). The majority of patients were white and accounts for 68 patients (73.1%). There were 14 African-American patients (15.1%), and the other ethnic groups comprised 11 patients (11.8%). 

Of the 93 patients, 82.8% patients had high grade (poorly or undifferentiated) histology; 13.9% patients presented with distant stage. Among the patients with known PSA value (*n* = 35), 80% of patients were found with elevated PSA, while the remaining 20% had a normal PSA value. 

Overall, the median duration of followup was 30 (range 0–238) months, and the median duration of follow-up for censored patients was 2.7 years. Finally, a total 39 of 93 (41.9%) patients died during the followup period.

### 3.3. Predictor of Receipt of Prostatectomy and Radiation Therapy

Cancer-directed surgery was performed in 40 (45%) patients; among them, 25 patients (26.9%) had radical prostatectomy. A total of 25 (26.9%) of the patients received primary radiation therapy, with adjuvant radiation following surgery in 5(5.4%) patients. 

In a logistic regression analysis, age, marital status, and tumor stage significantly correlated with radical prostatectomy; the patients who were older, unmarried and had advanced stage tumor were less likely to elect radical prostatectomy. In a separate analysis restricted to patients with local/regional disease, younger age and being married remained independent predictors of radical prostatectomy (Table 3).

Logistic regression analyses of factors associated with radiation therapy were also performed. African Americans were more likely to receive radiation therapy compared to the other races. Prostatectomy receipts were found to be less likely to receive radiation therapy (Table 4).

### 3.4. Long-Term Cancer-Specific Survival

**Figure 1:**
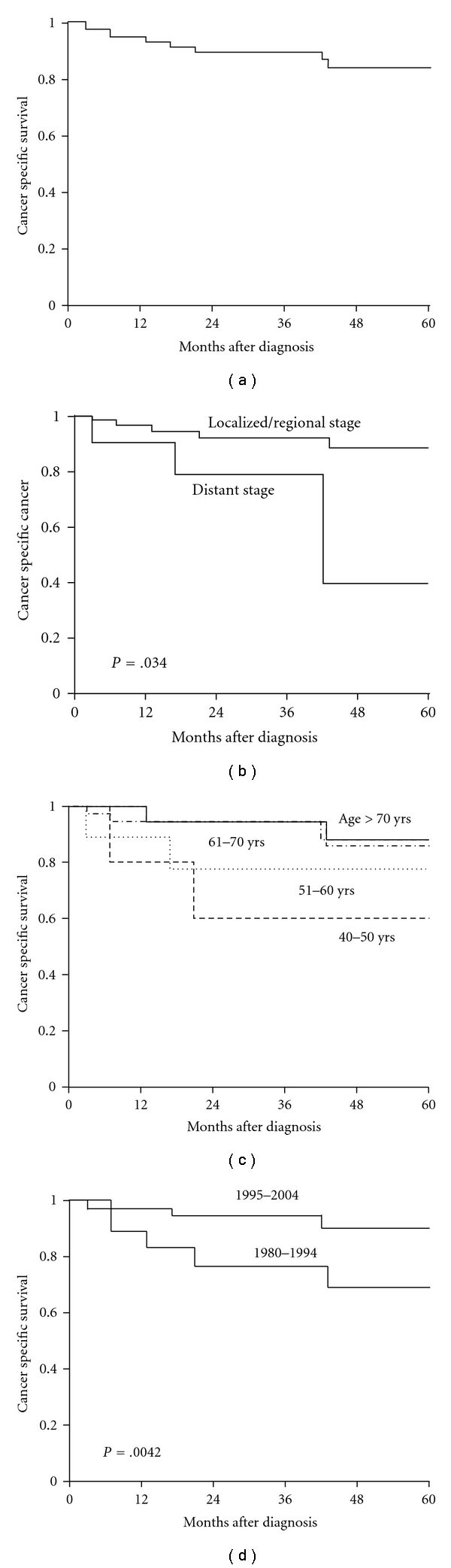
Kaplan-Meier survival curves for (a) cancer-specific survival of entire cohort, (b) cancer-specific survival by subgroups of different tumor stages, (c) cancer-specific survival by age subgroups, and (d) cancer-specific survival rate of patients by subgroups of different diagnosis year. *P*-value shown for log-rank test between two groups.

For cancer-specific survival analyses, the cases that were diagnosed at autopsy or on the basis of death certificates only as well as patients with multiple primaries were excluded. A total 81 patients were included in the survival analysis.  Table 5 presents the cancer-specific survival rates according to patient and tumor characteristics. Overall cancer-specific survival rates at 1-, 3-, and 5-year were 94.6%, 89.6%, and 83.8%, respectively (Figure 1(a)).The 1-, 3-, and 5-year cancer-specific survival rates for patients with local/regional stage tumor were 96.4%, 91.9%, and 88.3% and for patients with distant stage tumor were 90.0%, 78.8%, and 39.4%, respectively (Figure 1(b)). The median cancer-specific survival for patients who underwent prostatectomy was 100 months (95% CI 96–104). The 1-, 3-, and 5-year cancer-specific survival rates for patients who underwent prostatectomy were 94.7%, 89.2%, and 89.2%, respectively. There was a significant difference in survival between different age subgroups. The 1-, 3-, and 5-year cancer-specific survival rates for patients age >70 years old group were 94.7%, 94.7%, and 88% for patients ages between 40–50 years old were 80%, 60%, and 60%, respectively (Figure 1(c)). The 1-, 3-, and 5-year cancer-specific survival rates were significantly improved for patients who were diagnosed in later years (1995–2004) compared to those in earlier years (1980–1994) (Figure 1(d)). 

Table 6 presents the result of multivariate survival analyses using Cox proportional hazard model. After adjusting for the demographic, clinical, and treatment-related factors, younger age was identified as an independent predictor of poor survival in comparison to other older age subgroups (51–60 yrs versus 40–50 yrs, HR = 0.09; 61–70 yrs versus 40–50 yrs, HR = 0.04; >70 yrs versus 40–50 yrs, HR = 0.02). The tumor stage at diagnosis was another significant predictor of cancer-specific survival. Compared to patients with local/regional disease, patients with distant disease had nearly 11-fold increased risk of cancer-specific death from SRPC (*P* = .02). The other significant factor associated with survival was year of diagnosis (*P* = .01). Patients with SRPC diagnosed after1995 had significantly decreased cancer-specific death rate compared to those diagnosed before 1995 (HR = 0.12).

## 4. Discussion

Because of the rarity of SRPC, previously published information has been based on case series and single institutional experiences, which may not represent “real world” patients. Large, tertiary-care referral centers with mature local, regional, and national referral patterns may have a disproportionate number of advanced and recurrent tumors as well as a healthier population able to travel to these centers. This study takes advantage of the vast amount of data collected by the national SEER Program to examine the largest series of SRPC reported to date. We examine the incidence, natural history, predictors of utilization of prostatectomy, radiation therapy, and factors that affect the survival for SRPC by using the national population-based database. In this study, the total SRPC cases accounted for approximately 0.02% of primary prostate tumors included in the SEER database during study period. This incidence calculated in the SEER database is lower than reported in single institution studies [2, 5]. One of the potential explanations for these findings is referral bias. The patients with rare histology subtype are more likely to visit referral centers for a second opinion; compared with a community counterpart, pathologists from tertiary hospital and referral centers are more likely to have expertise in indentifying this rare subtype of histology. 

The optimal treatment strategy for this subtype of prostate cancer is unknown since there is no clinical trial specifically designed for SRPC. In this study, we indentified significant age-and racial-related disparities as an important factor for selecting prostatectomy or radiation therapy, which is consistent with previous findings [11, 13, 14]. For example, younger age was the strongest predictor for receiving radical prostatectomy rather than external beam radiotherapy (EBRT) and younger age as a predictor of aggressive local therapy, while older prostate cancer patients were often treated less aggressively [15, 16]. Efforts should be made to indentify high risk individuals for potential intervention in order to reduce these disparities. 

Consistent with single institution studies, the cancer-specific survival of SRPC is poor. In our study, the 3- and 5-year survival rates of SRPC were 89.6% and 83.8% (Table 5), which are significantly lower, in comparison with 5- and 10-year survival rates of 99.9% and 92%, respectively, in patients with prostate cancer as a whole [17].

Despite receiving more aggressive therapy, younger patients in this study had a poorer prognosis (Figure 1(c)). Controversy exists regarding the importance of patient age in disease behavior of prostate cancer. In several series, younger age at diagnosis has been correlated with more aggressive tumor types and subsequent mortality [18, 19]. Conversely, other studies have shown survival rates among younger patients to be equivalent or even superior to those of elderly patients [20–22]. Lin et al. [22] examined the association between age at diagnosis and grade, stage, treatment, and survival outcomes in men who were diagnosed during the era of PSA testing. Younger men were more likely to undergo prostatectomy, have lower grade cancer, and, as a group, to have better overall and equivalent cancer-specific survival at 10 years compared with older men. However, among men with high grade and locally advanced prostate cancer, the younger men had a poorer prognosis compared to older men. The subgroup of men with high grade and locally advanced prostate cancer described in Lin's study are very similar to our subjects. 82.8% of our entire study cohort (90% of the patients with known histology grade) had either poorly or undifferentiated histology. The findings collectively, from this study and others, support the view that prostate cancer is a heterogeneous disease, different subtypes may represent different diseases [22–25]. In practical terms, these findings suggest that better diagnosis and therapy of prostate cancer are likely to be achieved by investigating each subtype of prostate cancer separately rather than grouping them all together [25]. Our finding of subtype-related differences in survival has significant therapeutic implications with regard to patient selection, trial design, and therapy recommendations and warrants further study to offer this poor-prognosis group of men better preventative and therapeutic options.

Cancer incidence is known to be increased with age [12, 17]. Paradoxically, tumor growth and metastasis often occurred at a slower rate in aged human and animal populations. For example, tumors grow slower and metastasize less in old patients with cancer [26–28]. In experimental models, tumor growth has frequently been shown to be slower and to display a reduced aggressiveness in aged as compared to young animals [29]. The mechanisms responsible for these phenomenons have not yet been established. Decreased proliferative capacity [30], decline in growth factors with age [31], and age-related changes in antitumor immunity [32] have been suggested. Recently, an increased apoptotic cell death has also been linked to the reduced malignant behavior of tumors in the aged [33]. 

Currently, there is no literature addressing the impact of age on survival, specifically on SRPC. Our finding of age as an independent prognostic factor for this disease is interesting. The survival difference among different age groups might be explained by a difference in the biologic behavior of SRPC in the younger patients and elderly. Molecular research may provide additional insights into these questions. Similarly, breast cancer arising in young women is correlated with inferior survival and higher incidence of negative clinicopathologic features. A large-scale genomic analysis illustrates that breast cancer arising in young women is a unique biologic entity driven by unifying oncogenic signaling pathways [34]. Age-specific differences in oncogenic pathway dysregulation have also been investigated in patients with acute myeloid leukemia [35]. The age-related differential biological behavior of tumors also implies the necessity of a differential therapy for cancer patients of different ages. In fact older patients are now more frequently treated with radiation therapy than surgery modulated by projected patient longevity and biomarkers predicting cancer recurrence 7 years following initial biopsy or resected tumor specimen using Systems Pathology [35, 36]. 

Similar to the findings of increasing in 5-year survival in men with prostate cancer in post-PSA era, we also observed a significant improvement in the outcome of patients with SRPC during this period (Figure 1(d)). These improvements were consistent in all SEER stage groups and are likely due to widely application of PSA screening, early diagnosis, advance in local and systemic therapy, and increasing adoption of multidisciplinary prostate cancer care [14, 17]. 

Our findings should be interpreted with caution. First, this is a nonrandomized study; therefore, selection bias might have been present because patients undergoing surgery tend to be healthier. Although we adjusted for differences in demographic and tumor factors, residual confounding might still exist. Second, the pathological diagnoses in SEER were based on local pathologists' reports, and there was no central review of pathology reports. In addition, SEER data did not allow us to examine receipt of hormone or chemotherapy and patients' comorbidities. However, the analysis reported here attempted to overcome this data limitation by measuring prostate cancer-specific survival, rather than overall survival. Finally, the sample size in our study may still not be large enough to fully describe the factors that affect the incidence, treatment choice, and survival of this rare prostate cancer subtype. Our findings may not necessarily be generalized to patients with prostate adenocarcinoma as a whole.

Strengths of this study include the population-based design, larger sample size, and inclusion of a broad spectrum of hospitals in the analysis. Having a larger sample size is of particular importance for analysis of rare subtype prostate cancer such as SRPC, where it is nearly impossible for a single institution to collect enough cases to facilitate a meaningful analysis regarding prognostic factors.

## 5. Conclusions

Based on the analysis of a population-based database, we report the results of the largest series of SRPC in the literature. Patients with SRPC have a worse survival compared to patients with other types of prostate cancer. Younger SRPC patients have significantly lower cancer-specific survival rate compared to their older counterparts. Further study to elucidate the mechanism of the differential biological behavior of SRPC in relation to host age is planned. The development of new strategies and molecular-based therapeutic approaches for younger patients with this aggressive tumor is urgently needed.

## Figures and Tables

**Table 1:** Age-Adjusted Incidence Rate of Signet Ring Prostate Carcinoma.

	Age-adjusted incidence rate*	95% CI
All	0.0088	(0.0065–0.0116)
White	0.0077	(0.0054–0.0107)
Black	0.00187	(0.0085–0.0353)
Others	0.0081	(0.002–0.0206)

*Rates are per 100,000 populations (95% confidence interval) age-adjusted to year 2000 US standard population.

**Table 2:** Characteristics of 93 Patients with Signet Ring Prostate Carcinoma (Diagnosed Between January 1980 and December 2004).

Characteristics	Total patients *n* (%)
Age groups, *n* (%)	
40–50 yrs	5 (5.4)
51–60 yrs	13 (14.0)
61–70 yrs	30 (32.2)
>70 yrs	45 (48.4)
Race	
Black	14 (15.1)
White	68 (73.1)
Others	11 (11.8)
Married	
Yes	64 (68.8)
No	18 (19.4)
Unknown	11 (11.8)
Grade	
Moderately-differentiated	9 (9.7)
Poorly-differentiated	74 (79.6)
Undifferentiated	3 (3.2)
Unknown	7 (7.5)
PSA	
Above normal	29 (31.2)
Normal	6 (6.5)
Unknown	58 (62.4)
Stage	
Local/regional	72 (77.9)
Distant	13 (13.9)
Unknown	8 (8.6)
Year of diagnosis	
1980–1994	22 (23.7)
1995–2004	71 (76.3)
Prostatectomy	
Yes	25 (26.9)
No	68 (73.1)
Radiation	
Yes	25 (26.9)
No	66 (71.0)
Unknown	2 (2.1)

PSA: prostate specific antigen.

**Table 3:** Multivariate Analyses of Factors Associated with Receipt of Radical Prostatectomy in Patients with Localized/Regional Stage Signet Ring Prostate Carcinoma.

Characteristics	Group	OR	95% CI	*P*-value
Age	Continuous	0.80	0.71–0.90	<.001
Ethnicity	White	1.00		
	Black	3.85	0.39–38.0	0.25
	Others	1.79	0.22–14.4	0.58
Marital status	Married	1.00		
	No	0.12	0.02–0.85	0.03
Year of Diagnosis	1980–1994	1.00		
	1995–2004	0.62	0.13–2.97	0.55
Radiation	No	1.00		
	Yes	0.10	0.01–0.92	0.04

OR: odds ratio; CI: confidence interval.

**Table 4:** Multivariate Analyses of Factors Associated with Receipt of Radiation in Patients with Signet Ring Prostate Carcinoma.

Characteristics	Group	OR	95% CI	*P*-value
Age	Continuous	0.97	0.92–1.03	0.34
Ethnicity	White	1.00		
	Black	8.67	1.66–45.36	0.01
	Others	0.44	0.04–4.54	0.49
Year of Diagnosis	1980–1994	1.00		
	1995–2004	1.27	0.35–4.67	0.72
SEER stage	Local/regional	1.00		
	Distant	0.30	0.06–1.51	0.15
	Unstaged	0.31	0.03–3.0	0.31
Marital status	Married	1.00		
	No	0.47	0.14–1.62	0.23
Prostatectomy	No	1.00		
	Yes	0.06	0.01–0.44	0.006

OR: odds ratio; CI: confidence interval.

**Table 5:** 1-, 3-, and 5-year cancer-specific Survival of Patients with Signet Ring Prostate Carcinoma According to Demographic and Clinical Characteristics.

Characteristics		Cancer-Specific Survival Rate (%)		
		1-year	3-year	5-year
Overall patients		94.6	89.6	83.8
Age subgroups	40–50 yrs	80	60	60
	51–60 yrs	88.8	77.8	77.8
	61–70 yrs	94.4	94.4	85.9
	>70 yrs	94.7	94.7	88
SEER stage	Local/regional	96.4	91.9	88.3
	Distant	90.0	78.8	39.4
	unstaged	87.5	87.5	87.5
Prostatectomy	No	94.5	80.9	80.9
	Yes	94.7	89.2	89.2
Year	1980–1994	88.9	76.6	68.9
	1995–2004	96.5	94.3	89.8

**Table 6:** Multivariate Analyses of Factors Associated with Cancer-Specific Mortality in Patients with Signet Ring Prostate Carcinoma.

Characteristics	Group	HR	95% CI	*P*-value
Age	40–50 yrs	1.00		
	51–60 yrs	0.09	0.007–1.15	0.06
	61–70 yrs	0.04	0.003–0.58	0.02
	>70 yrs	0.02	0.001–0.37	0.01
Ethnicity	White	1.00		
	Black	2.32	0.23–23.5	0.47
	Others	2.51	0.29–21.6	0.40
SEER Stage	Local/regional	1.00		
	Distant	10.9	1.44–82.09	0.02
	Unstaged	5.23	0.70–39.3	0.11
Marital status	Married	1.00		
	No	0.11	0.01–0.93	0.04
Diagnosis year	1980–1994	1.00		
	1995–2004	0.12	0.02–0.62	0.01
Prostatectomy	No	1.00		
	Yes	0.57	0.07–4.40	0.59
Radiation	No	1.00		
	Yes	0.28	0.03–2.52	0.25

HR: Hazard ratio; CI: Confidence interval.
